# Supplementary material for: clusTransition: An R package for monitoring transition in cluster solutions of temporal datasets
Source: PLoS One. 2022 Dec 15;17(12):e0278146. doi: 10.1371/journal.pone.0278146 (PMC9754593; doi:10.1371/journal.pone.0278146)
Supplement: Attachment — Submitted filename: Response to Reviewers.docx [file pone.0278146.s002.docx]

**Response to Reviewers**

Thank you for giving me the opportunity to submit a revised draft of my manuscript titled
clusTransition: An R Package for Monitoring Transition in Cluster Solutions of Temporal
Datasets to PLOS ONE. We appreciate the time and effort that you and the reviewers have
dedicated to providing your valuable feedback on my manuscript. We are grateful to the
reviewers for their insightful comments on my paper. We have highlighted the changes within
the manuscript.

Here is a point-by-point response to the reviewers’ comments and concerns.

Reviewer #2: The author has responded to all my comments in the previous review round sufficiently. I have one more minor comment. Please consider merging the "Related works" section into the introduction since this section is too small to be a stand-alone section in the manuscript.

**Response: Corrected**
